# The impact of mass vaccination policy and control measures on lumpy skin disease cases in Thailand: insights from a Bayesian structural time series analysis

**DOI:** 10.3389/fvets.2023.1301546

**Published:** 2024-01-05

**Authors:** Veerasak Punyapornwithaya, Orapun Arjkumpa, Noppawan Buamithup, Chalita Jainonthee, Roderick Salvador, Katechan Jampachaisri

**Affiliations:** ^1^Research Center for Veterinary Biosciences and Veterinary Public Health, Faculty of Veterinary Medicine, Chiang Mai University, Chiang Mai, Thailand; ^2^Veterinary Public Health and Food Safety Centre for Asia Pacific (VPHCAP), Faculty of Veterinary Medicine, Chiang Mai University, Chiang Mai, Thailand; ^3^Department of Veterinary Biosciences and Veterinary Public Health, Faculty of Veterinary Medicine, Chiang Mai University, Chiang Mai, Thailand; ^4^The 4^th^ Regional Livestock Office, Department of Livestock Development, Khon Kaen, Thailand; ^5^Department of Livestock Development, Bangkok, Thailand; ^6^College of Veterinary Science and Medicine, Central Luzon State University, Science City of Muñoz, Nueva Ecija, Philippines; ^7^Department of Mathematics, Faculty of Science, Naresuan University, Phitsanulok, Thailand

**Keywords:** lumpy skin disease, intervention, mass vaccination, Bayesian structural time series, causal impact, Thailand

## Abstract

**Introduction:**

In 2021, Thailand reported the highest incidence of lumpy skin disease (LSD) outbreaks in Asia. In response to the widespread outbreaks in cattle herds, the government's livestock authorities initiated comprehensive intervention measures, encompassing control strategies and a national vaccination program. Yet, the efficacy of these interventions remained unevaluated. This research sought to assess the nationwide intervention's impact on the incidence of new LSD cases through causal impact analysis.

**Methods:**

Data on weekly new LSD cases in Thailand from March to September 2021 was analyzed. The Bayesian structural time series (BSTS) analysis was employed to evaluate the causal relationship between new LSD cases in the pre-intervention phase (prior to the vaccination campaign) and the post-intervention phase (following the vaccination campaign). The assessment involved two distinct scenarios, each determined by the estimated effective intervention dates. In both scenarios, a consistent decline in new LSD cases was observed after the mass vaccination initiative, while other control measures such as the restriction of animal movement, insect control, and the enhancement of the active surveillance approach remained operational throughout the pre-intervention and the post-intervention phases.

**Results and discussion:**

According to the relative effect results obtained from scenario A and B, it was observed that the incidence of LSD cases exhibited reductions of 119% (95% Credible interval [CrI]: −121%, −38%) and 78% (95% CrI: −126, −41%), respectively. The BSTS results underscored the significant influence of these interventions, with a Bayesian one-sided tail-area probability of *p* < 0.05. This model-based study provides insight into the application of BSTS in evaluating the impact of nationwide LSD vaccination based on the national-level data. The present study is groundbreaking in two respects: it is the first study to quantify the causal effects of a mass vaccination intervention on the LSD outbreak in Thailand, and it stands as the only endeavor of its kind in the Asian context. The insights collected from this study hold potential value for policymakers in Thailand and other countries at risk of LSD outbreaks.

## 1 Introduction

Lumpy skin disease (LSD) is a significant transboundary disease affecting the bovine population causing substantial economic losses in cattle industries across countries (1). The disease is caused by the lumpy skin disease virus (LSDV) belonging to the *Capripoxvirus* genus, of the *Poxviridae* family (2). Its primary symptoms include nodular lesions on the skin and mucous membranes, enlargement of lymph nodes, and elevated body temperature (3, 4). Affected cattle may experience weight loss, decreased milk production, and deteriorated skin quality, all contributing to economic losses. While the morbidity rate of LSD is moderate, its mortality rate remains low (5). The disease primarily spreads through insect vectors (1) but can be effectively controlled and prevented with vaccines (6–8). The primary strategies for managing and eradicating LSD are restricting animal movement and mass vaccination (5, 9). Given its potential for swift spread and significant economic impact, the World Organization for Animal Health (WAOH) classifies LSD as a notifiable disease, necessitating reporting of its occurrences (5). While LSD is endemic in Africa (2), it reached the Middle East in 2012, Europe in 2015, and had outbreaks in Russia between 2017 and 2019. The disease further spread to the South and East Asia between 2019–2020 (3, 10, 11). From 2020 onwards, several Southeast Asian countries reported multiple LSD outbreaks (12–14) with Thailand recording the highest incidence in 2021 (15, 16).

Thailand reported the first LSD outbreak in cattle farming areas in the northeastern region in April 2021 (17). Within a few months, the disease had spread nationwide (18–20). In response, starting from mid-April 2021, authorities in charge of livestock implemented various control measures including regulating cattle movement, disinfecting affected areas, managing insect vectors, treating affected animals, closing live cattle markets, and instituting active surveillance to identify infected premises. Local veterinarians and livestock officers across the country took on the responsibility to control LSD outbreaks in their respective areas to contain the swift dissemination of lumpy skin disease viruses from outbreak areas to other regions (18, 21, 22). Additionally, a public awareness campaign was initiated to spread information about preventive measures (18). This campaign involved in-person meetings between livestock authorities and farmers, along with an online website that regularly updated the status of LSD outbreaks in the country. Despite these efforts, there was a continuous rise in new LSD cases at the national level (23). In June 2021, the government initiated a mass vaccination campaign with the primary goal of controlling nationwide LSD outbreaks (24, 25). After the implementation of mass vaccination, the number of new LSD cases appeared to continuously decrease (18). Nonetheless, there has been no study conducted to assess the impact of this implementation.

Interrupted time series (ITS) analysis is increasingly used to evaluate the effectiveness of interventions, ranging from clinical treatments to broad public health policies (26–29). This analytical method is commonly employed to assess the effectiveness of large-scale population healthcare interventions. For example, ITS has been applied to evaluate large-scale health interventions initiated by the Australian government (27). Similarly, ITS has been instrumental in assessing the causal impact of lockdown measures on COVID-19 in both China (30) and Brazil (31) as well as the influence of social distancing on the COVID-19 epidemic in the United States (32) and Europe (33). Notably, the term “causal impact” is frequently mentioned in research publications that use ITS analysis to determine the effects of specific interventions (34–36).

Various methods of ITS have been proposed such as ordinary least square regression, generalized least square, restricted maximum likelihood, and autoregressive integrated moving average (29, 37–40). Within a Bayesian framework, Bayesian structural time series (BSTS) model is widely used. This quasi-experimental model is designed to assess the impact of an intervention by predicting a counterfactual time series, which represents the anticipated outcome in the absence of the intervention. The counterfactual is determined through an evaluation of the time series data. Ultimately, the assessment of impact involves comparing the observed time series during the intervention period to the counterfactual scenario generated by the model, thereby determining the difference (41–43). This approach has been adopted in multiple studies (35, 43–45). A distinct advantage of BSTS over alternative models is its counterfactual approach, while other models predominantly aim to identify overarching trend changes (46). Thus, this study attempted to determine the impact of the mass vaccination intervention using the BSTS method. In this regard, we estimated the number of new LSD cases under the premise that no mass vaccination campaign took place. These estimates were then compared with the actual figures from scenarios where mass vaccinations were conducted.

While many studies in Asia provide valuable insights into the epidemiology of LSD, there is a limited number of research focusing on large-scale national efforts to control LSD outbreaks in this region. Hence, this study aims to determine the effectiveness of the vaccination campaign implemented to control LSD outbreaks in Thailand using the BSTS approach.

## 2 Materials and methods

### 2.1 LSD outbreaks and intervention policy

**Figure 1:**
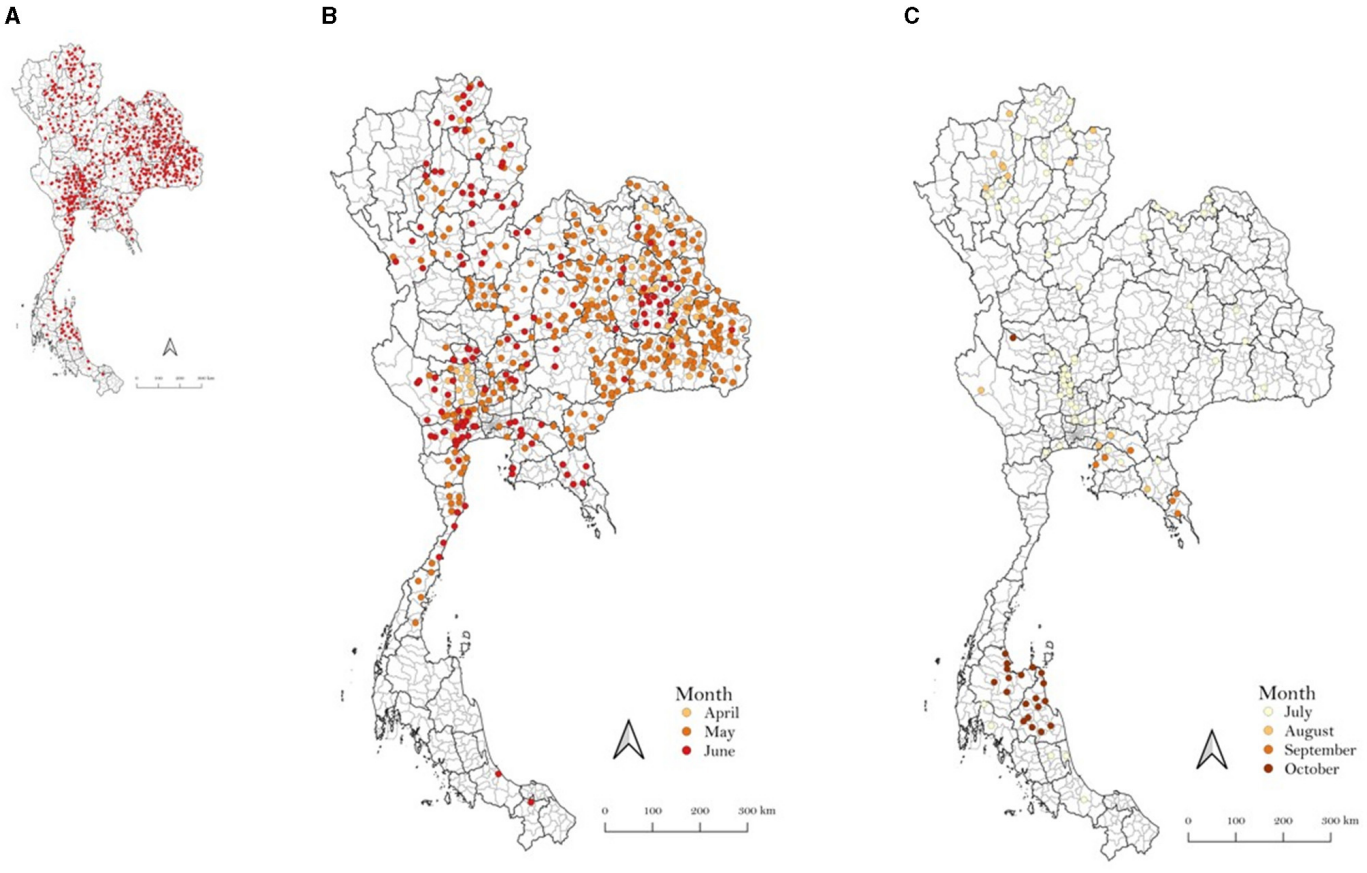
Outbreak areas of lumpy skin disease in Thailand in 2021 including the outbreaks from April to December **(A)**, April to June **(B)**, and July to December **(C)**.

**Figure 2:**
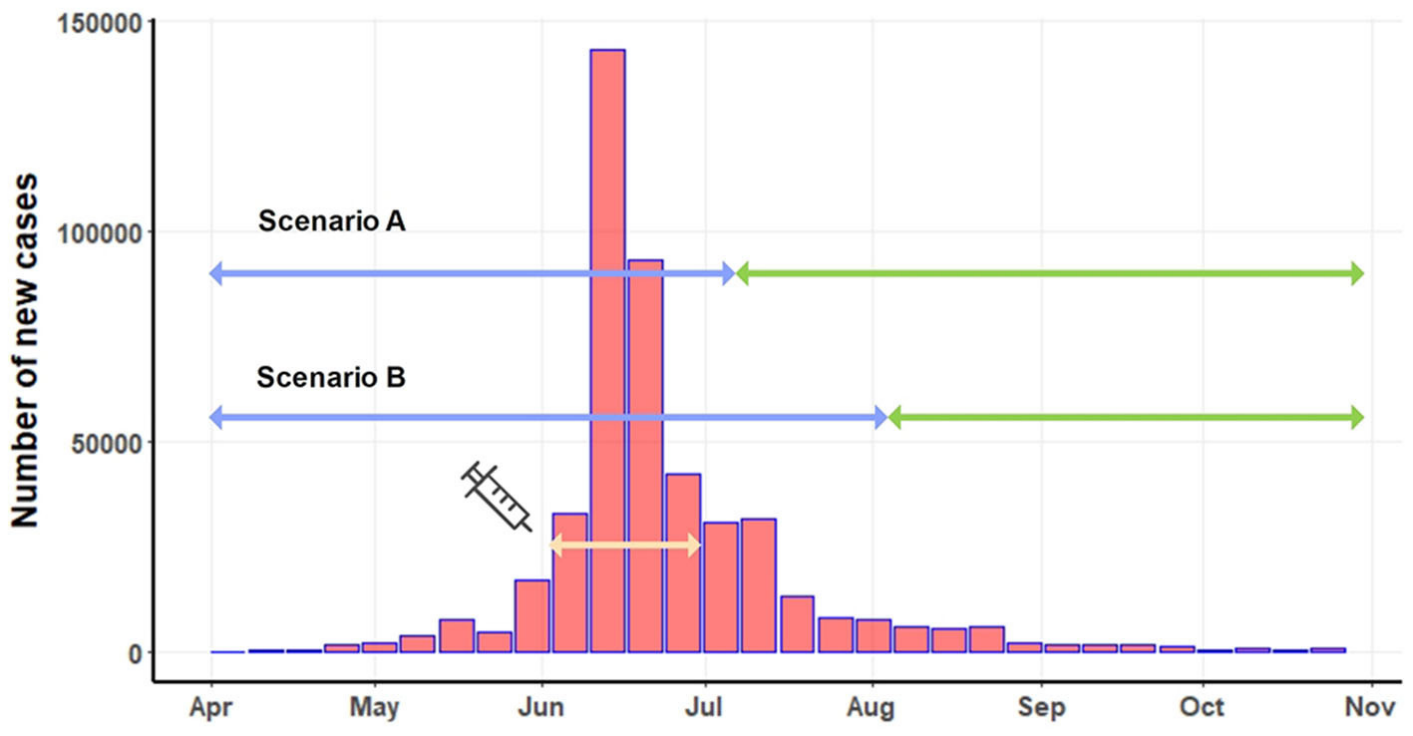
Number of new LSD cases on a weekly basis. The light-yellow line depicts the period of the mass vaccination campaign. Based on two scenarios, the blue line indicates the pre-intervention period, and the green line represents the post-intervention period.

In 2021, LSD outbreaks were reported in numerous areas across Thailand by the livestock authorities of the Department of Livestock Development (DLD). The outbreaks were confirmed through clinical diagnosis and by testing samples from LSD-affected animals using the PCR technique at the DLD laboratory, as described in previous studies (17–19). Figure 1 illustrates the LSD outbreak areas in Thailand, indicating a widespread occurrence throughout the country. Additionally, the number of new LSD cases from April to November 2021 is illustrated in Figure 2. For additional details, the graph presenting the number of new LSD cases on a daily basis is included in Supplementary Figure S1.

This study utilized the reporting of the number of LSD-affected cattle from the national livestock system organized by DLD. Initially, provincial livestock officers provided the reports, having received information from district-level officers investigating LSD outbreaks on farms. Subsequently, using the data collected by these livestock officers, the total number of new LSD cases was calculated. The dataset utilized in the present study includes the number of new LSD cases on a weekly basis, spanning from April to November 2021.

The mass vaccination campaign was implemented in June 2021. A total of 360,000 doses of live attenuated homologous vaccines were administered to cattle in high-risk areas for LSD outbreaks across the country (18). The effectiveness of the mass vaccination campaign, initiated in early June, couldn't be expected to manifest instantly. Implementing such a nationwide campaign involves multiple considerations to determine its effective date. Firstly, numerous administrative tasks, including paperwork, vaccine transportation, and identification of target farmers, must be completed to distribute the vast quantity of vaccine doses throughout the country. Secondly, time was allocated for the registration and verification of farm owners, as well as the actual vaccination procedures in different regions. Thirdly, post-vaccination, immediate immunity is not conferred to the cattle (7, 47). Research indicates that antibodies typically emerge within 15 days and peak around 30 days after vaccination (5). Taking these factors into consideration, we propose two start dates at which the majority of the vaccinated cattle population would attain sufficient immunity to the disease: 45 days (July 15) and 60 days (July 30) post-June 1, identified as scenario A and scenario B. Under scenario A, the pre-intervention period extended from April 1 to July 15, while the pre-intervention period for scenario B covered April to July 30. The post-intervention periods for scenario A and scenario B were July 15 to November 31 and August 1 to November 31, respectively (Figure 2).

It is crucial to note that control measures, such as restrictions on animal movement, closure of live cattle markets, insect control, disinfection on farms, active surveillance for LSD cases, and heightened public awareness, have been consistently enforced since April 1. These measures were applied throughout both the pre-intervention and post-intervention periods.

### 2.2 Bayesian structural time series models

The Bayesian methodologies based on STSM, termed as BSTS models has been proposed by Brodersen and Hauser (41). These BSTS models are state-space models tailored for time series data, which can be defined through a set of equations as described in (41, 48, 49):


(1) $$\begin{array}{l} y_{t}=z_{t}^{T}\alpha_{t}+\varepsilon_{t} \end{array}$$

(2) $$\begin{array}{l} \alpha_{t+1}=T_{t}\alpha_{t}+R_{t}\eta_{t} \end{array}$$

where $\varepsilon_{t}\sim N\left(0,\sigma_{t}^{2}\right)$ and η_*t*_~*N*(0, *Q*_*t*_) are independent of all unknown data. Equation 1 serves as the observation equation that links the observed data *y*_*t*_ to the latent state vector α_*t*_. Equation 2 acts as the state equation detailing the progression of the vector α_*t*_ over time. The term *y*_*t*_ denotes the observed value, *Z*_*t*_ represents a d-dimensional output vector, and *T*_*t*_ is a *d*×*d* transition matrix. *R*_*t*_ is a *d*×*q* matrix, while ε_*t*_ signifies the observation error with a white noise variance α_*t*_. The term η_*t*_ is a q-dimensional system error with a *q*×*q* state-diffusion matrix *Q*_*t*_, where *q* ≤ *d*.

The BSTS approach is utilized to learn these parameters. Subsequently, the Markov chain Monte Carlo (MCMC) technique in conjunction with a Gibbs sampler, is used for posterior inference (50). The causal effect is estimated by contrasting the counterfactual (predicted) values with the actual LSD case numbers during the post-intervention phase (49). A comprehensive description of the statistical models and graphical procedures can be found in prior studies (41, 51).

The R statistical software, with the “*Causallmpact*” package (41), was utilized to determine the causal effect and assess the statistical significance of the intervention using the BSTS approach. The CausalImpact package provides an intuitive interface that conceals the typical intricacies linked with Bayesian analysis such as normalization, setting priors and estimation. It seamlessly integrates with the bsts package for the actual model fitting process. In the initial step, the BSTS model predicted the number of new LSD cases under the assumption that the mass vaccination campaign was not implemented (counterfactual). In the second step, the evaluation of the difference between the predicted and actual number of new LSD cases was conducted to quantify the impact of the mass vaccination. Subsequently, point effects, absolute effects, and relative effects, each accompanied by their 95% credible intervals (CrIs), were derived by comparing the predicted and actual death trends across 1,000 MCMC iterations, which is a default value.

The results from the BSTS process in the “CausalImpact” package analysis provide three graphical representations: original, pointwise, and cumulative. The graph consists of actual observed values and the predicted values during the post-intervention period which represents the causal effect of the intervention. The difference the predicted values to the actual values corresponding to an impact of the intervention can be displayed per time-point and cumulative using pointwise and cumulative graphs. Furthermore, in the analysis, the BSTS model makes the assumption that the relationship between interventions and the time series, which was established during the pre-period, continues to be stable throughout the post-period (50).

## 3 Results

For scenario A (Table 1), the post-intervention period saw an average of 2,941 new LSD cases. In an event without intervention, this number was projected to be 25,970. The difference between the predicted and actual values amounts to −23,029 in terms of absolute effect, indicating a 119% (95% CrI: −121%, −38%) decrease in relative effect. Additionally, the cumulative count of new LSD cases stood at 58,811, whereas without the intervention, it was projected to be 519,401. This translates to a relative decrease of 119%. The likelihood of this effect occurring by mere chance is minimal, with a Bayesian one-sided tail-area probability of *p* = 0.04, indicating statistical significance.

**Table 1:** Summary of actual, predicted, absolute and relative effects from a Bayesian structural time series analysis based on scenario A.

	**Average**	**Cumulative**
Actual	2,941	58,811
Prediction (s.d.)	25,970	519,401
95% CrI	[−31, 52,844]	[−622, 1,056,873]
Absolute effect (s.d.)	−23,029	−460,590
95% CrI	[−50,000, 2972]	[−600,000, 59,433]
Relative effect (s.d)	−119% (689%)	−119% (689%)
95% CrI	[−121%, −38%]	[−121%, −38%]

Posterior tail-area probability p: 0.04. Posterior prob. of a causal effect: 95.486%. s.d, standard deviation; CrI, Credible interval.

For scenario B (Table 2), in the absence of mass vaccination, the predicted number of cases was 24,273. The actual cases were only 2,085 cases, signifying a relative effect of the intervention, which equates to a 78% (95% CrI: −126, −41%) decrease (*p* = 0.04).

**Table 2:** Summary of actual, predicted, absolute and relative effects from a Bayesian structural time series analysis based on scenario B.

	**Average**	**Cumulative**
Actual	2,085	37,523
Prediction (s.d.)	24,273 (13,118)	436,915 (236,118)
95% CrI	[−2,001, 52,880]	[−36,019, 951,836]
Absolute effect (s.d.)	−22,188 (13,118)	−399,392 (236,118)
95% CrI	[−50,795, 4,086]	[−914,313, 73,542]
Relative effect (s.d.)	−78% (331%)	−78% (331%)
95% CrI	[−126%, −41%]	[−126%, −41%]

Posterior tail-area probability p: 0.04. Posterior prob. of a causal effect: 95.4%. s.d., standard deviation; CrI, Credible interval.

**Figure 3:**
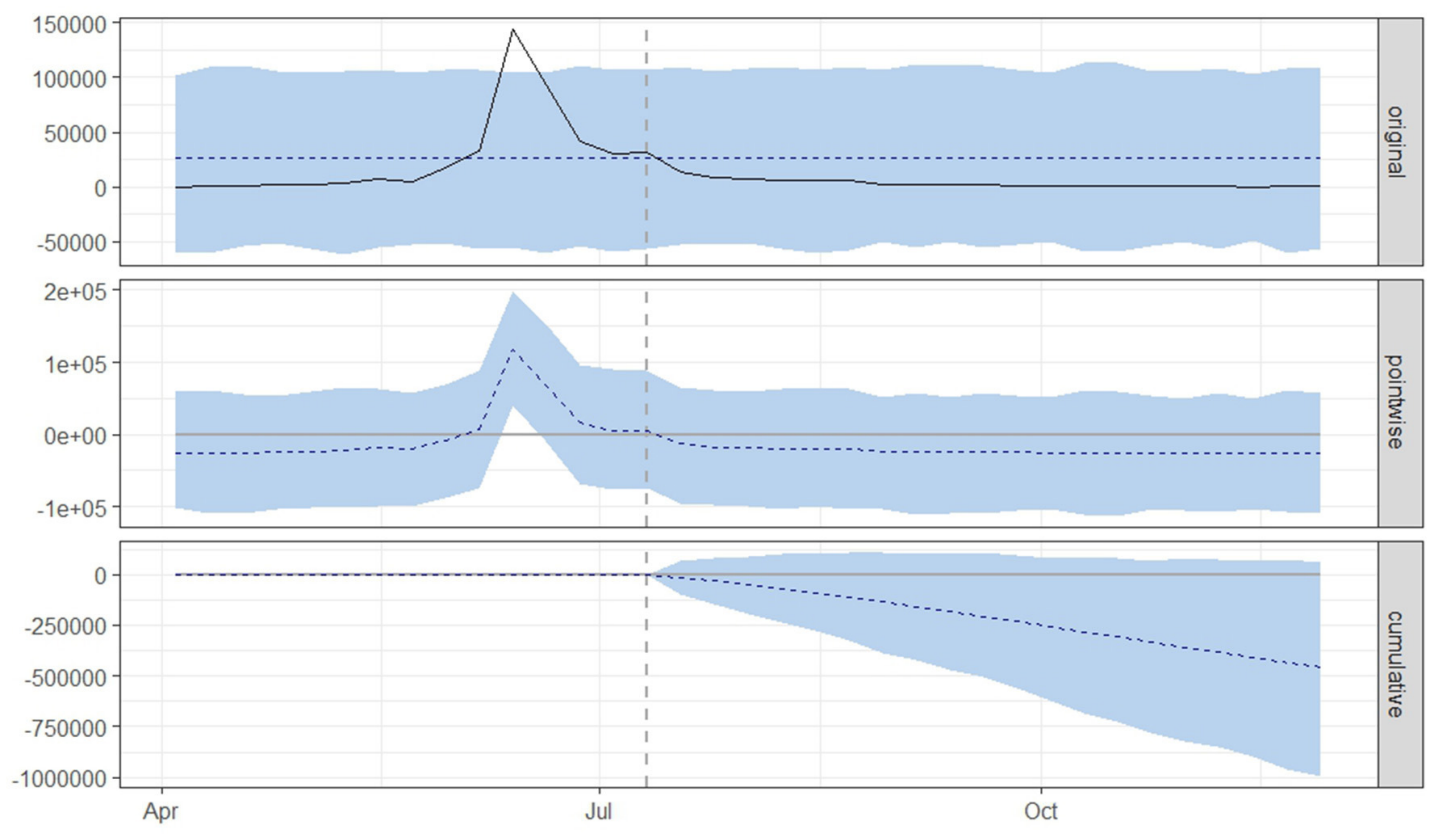
Graphical representation based on a causal impact analysis pertaining to the implementation of a nationwide mass vaccination campaign aimed to control the outbreak of lumpy skin disease, as per scenario A. The graph includes the original data, pointwise and cumulative effects of the intervention.

**Figure 4:**
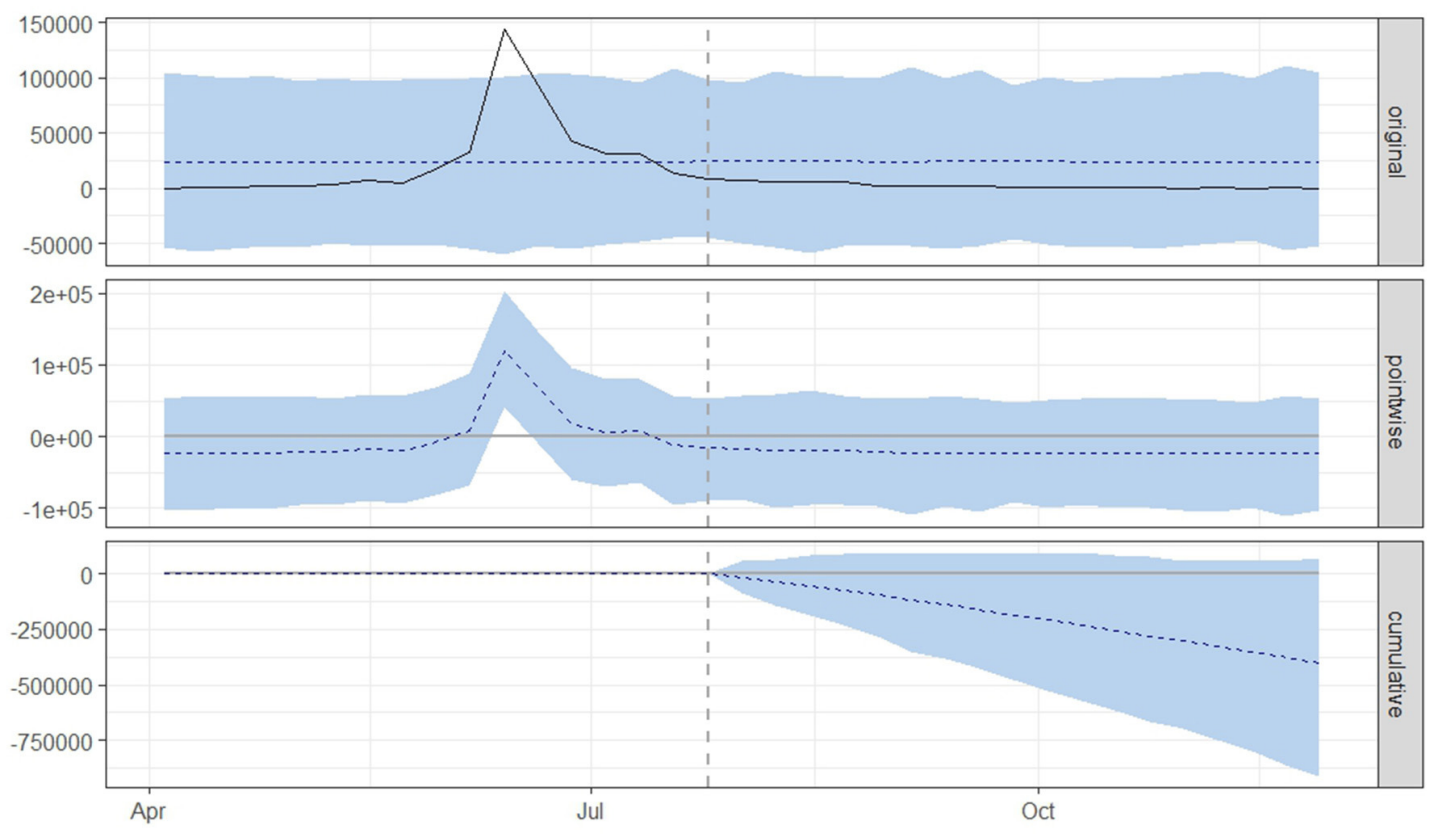
Graphical representation based on the causal impact analysis pertaining to the implementation of nationwide mass vaccination campaign to control the outbreak of lumpy skin disease, as per scenario B. The graph includes the original data, pointwise and cumulative effects of the intervention.

Figures 3, 4 displays three panels that visually represent the results. The first panel showcases both the actual data and a counterfactual projection for the post-treatment phase. The second panel highlights the pointwise causal effect, evident from the difference between the observed data and the hypothetical forecasts. The third panel captures the cumulative impact of the intervention by aggregating the pointwise effects from the second panel.

From both scenario A (Figure 3) and scenario B (Figure 4), it is evident that the interventions influenced the number of new LSD cases. The top panel anticipated a consistent number of new LSD cases (represented by the dashed line) if no intervention took place. However, the actual figures (depicted by the solid line) reveal a decline in new LSD cases post-intervention. The middle panel's pointwise causal effect displays negative values, representing the divergence between the actual numbers and the counterfactual predictions. The cumulative influence of the intervention is delineated in the bottom panel.

## 4 Discussion

The introduction of control measures and a mass vaccination policy in Thailand represented a significant effort to curb the LSD epidemic. Through a causal impact analysis employing the BSTS methodology, this study underscores the efficacy of these interventions in reducing the number of LSD cases in Thailand.

Following the confirmation of the initial LSD outbreak in April (17), a series of control measures were rolled out to limit the spread of the disease. However, between May and June, there was a consistent rise in new LSD cases (18). This surge could be attributed to the vulnerability of naive herds lacking immunity to the disease, given that the LSD vaccine had never been used in Thailand before the discovery of the first LSD outbreak. The rapid spread of LSD could also be linked to challenges in controlling insect vectors (19, 20) and unauthorized animal movements (18).

In the context of an LSD outbreak, the most effective control measure involves widespread cattle immunization coupled with restrictions on animal movement (2). Predominantly, commercially available vaccines for LSD are live attenuated formulations derived from LSDV strains, sheep pox virus (SPPV), or goat pox virus (GTPV) (6, 16). Specifically, live attenuated LSDV vaccines, commonly referred to as Neethling vaccines, have been widely used in cattle across various countries. It is acknowledged that these vaccines exhibit notable efficacy, providing robust protection for cattle against virulent field LSDV strains (6). In line with the government control strategy, mass vaccination of live attenuated homologous LSD vaccines was conducted in multiple areas across Thailand. The mass vaccination was implemented following the ring vaccination strategy, with a focus on administering LSD vaccines to control outbreaks, especially in high-risk areas. This typically included neighboring regions within a 5–50 km radius of the LSD outbreak areas (18, 25). The effectiveness of mass vaccination efforts was notably evident when comparing actual values to projection values from the BSTS model, assuming no vaccination. The observed decrease in the number of cases by more than 75%, as predicted by the two scenarios, serves as compelling evidence of the success of mass vaccination initiatives. It is important to note that our findings align with prior studies highlighting the efficacy of mass vaccination in controlling LSD outbreaks. In South-Eastern Europe, a regional mass vaccination campaign using a homologous LSD vaccine covered over 1.8 million bovines, proving instrumental in managing LSD in that region (9). Similarly, the deployment of Neethling vaccines was recognized as an effective measure against LSD in Israel in 2012 (52) and in the Balkans from 2015 to 2017 (53).

A noteworthy control measure implemented by DLD was the establishment of a reporting system for LSD outbreaks. Local livestock authorities, including district and provincial livestock offices, actively participated by providing daily updates on the situation. Once these reports are reviewed and verified, DLD consolidates the data and publishes it on its website for public access. This online resource offers biweekly and monthly overviews of the LSD outbreak status in Thailand (https://sites.google.com/view/dldlsd/home). Through this mechanism, farmers and other stakeholders are consistently updated on the nation's LSD situation. Moreover, the proactive involvement of local livestock authorities is crucial in managing the LSD outbreak. They diligently identify LSD cases in herds by purposefully visiting farms in their respective localities (18, 54).

Following the nationwide mass vaccination, there was a noticeable decline in new LSD cases in Thailand toward the end of 2021 (22). However, some LSD cases were still reported in 2022 (55). One plausible reason for the persistence of a few new LSD cases could be the non-vaccination of certain cattle, such as the young ones or those in specific herds during the campaign. Additionally, farmers might have faced challenges in procuring vaccines for newborn animals post-campaign. Therefore, we advocate for the continuation of the vaccination process to ensure sustained immunity against LSD in Thai cattle. Given the limited research on the longevity of cattle immunity against LSD post-vaccination in Thailand, annual vaccination might be a prudent approach. This could be funded either by farmers and stakeholders directly or through government subsidies. It's imperative to achieve comprehensive vaccination coverage, and farmers should be motivated to persist with the vaccination regimen for multiple years. This should be overseen by livestock authorities and policymakers to prevent a decline in vaccination practices, as witnessed in Israel, which could potentially lead to a resurgence in LSD outbreaks (56). Strengthening LSD surveillance in cattle herds is also crucial. Livestock authorities should guide farmers on vigilant monitoring for new LSD cases within their herds. In the event of a suspected case, immediate notification to livestock authorities is essential, emphasizing the need for a swift response from these authorities to the farmers' concerns.

This study utilized univariate time series data for BSTS analysis, aligning with methodologies employed in previous studies (40, 43). In this approach, individual characteristics, such as the breed and age of animals, were not integrated into the BSTS models. Therefore, it is important to note that the estimations produced by the BSTS models in this study were solely contingent on the number of new LSD cases presented in the form of time series data. Furthermore, the interpretation of the results, which implies a decrease in the number of new LSD cases during the post-intervention period, should not be exclusively attributed to the mass vaccination campaign. Rather, it should be viewed as reflective of the impact of the mass vaccination campaign, taking into account the sustained implementation of other control measures both before and after the vaccination campaign.

This study comes with certain limitations. The reported number of LSD cases may underestimation of the actual count due to under-reporting issue. This could arise from herd owners not reporting outbreaks, occurrences in remote areas beyond regular surveillance, or certain LSD cases developing after the survey period. However, the impact of this limitation may not be significant, given the proactive approach in detecting LSD outbreaks by livestock authorities and the high level of cooperation from farmers. Furthermore, some farmers might purchase vaccines from private sectors and vaccinate their cattle independently. While the number of farmers engaging in this practice may not be high compared to those receiving government-provided vaccines at no cost, it is a situation that should be addressed.

## 5 Conclusion

This study represents the first application of the BSTS methodology to evaluate the impact of a government-led mass vaccination campaign against the LSD epidemic, drawing from national-level data. The methodologies employed in this research offer a deeper understanding of how established statistical tools can be used to determine the causal effects of intervention policies on large-scale LSD outbreaks. The findings from this investigation highlight the efficacy of the intervention strategy in mitigating LSD outbreaks. Notably, Thailand stands out as the only Asian country to have implemented a nationwide mass vaccination with extensive coverage. Consequently, the strategies adopted by Thai livestock authorities and stakeholders concerning mass vaccination and control measures could serve as valuable references for other countries at risk to a widespread LSD epidemic.

## Data availability statement

The datasets presented in this article are not readily available because the dataset used in this research is sourced from the Department of Livestock Development (DLD) in Thailand. Requests to access the datasets should be directed to foreign@dld.go.th.

## Ethics statement

This study is based on secondary data sourced from the Department of Livestock Development, Ministry of Agriculture and Cooperatives, Thailand. As our research exclusively employs pre-existing data and does not involve direct interactions with live animals, there is no requirement for ethical approval related to animal use.

## Author contributions

VP: Conceptualization, Data curation, Formal analysis, Funding acquisition, Methodology, Project administration, Software, Supervision, Validation, Visualization, Writing—original draft, Writing—review & editing. OA: Data curation, Investigation, Methodology, Resources, Validation, Visualization, Writing—review & editing. NB: Data curation, Investigation, Methodology, Resources, Validation, Writing—review & editing. CJ: Data curation, Validation, Visualization, Writing—review & editing. RS: Methodology, Validation, Visualization, Writing—original draft, Writing—review & editing. KJ: Conceptualization, Methodology, Project administration, Software, Supervision, Writing—review & editing.
