# Supplementary material for: The impact of mass vaccination policy and control measures on lumpy skin disease cases in Thailand: insights from a Bayesian structural time series analysis
Source: Front Vet Sci. 2024 Jan 5;10:1301546. doi: 10.3389/fvets.2023.1301546 (PMC10797105; doi:10.3389/fvets.2023.1301546)
Supplement: Supplementary file 1 [file Image_1.DOCX]

**Supplementary**


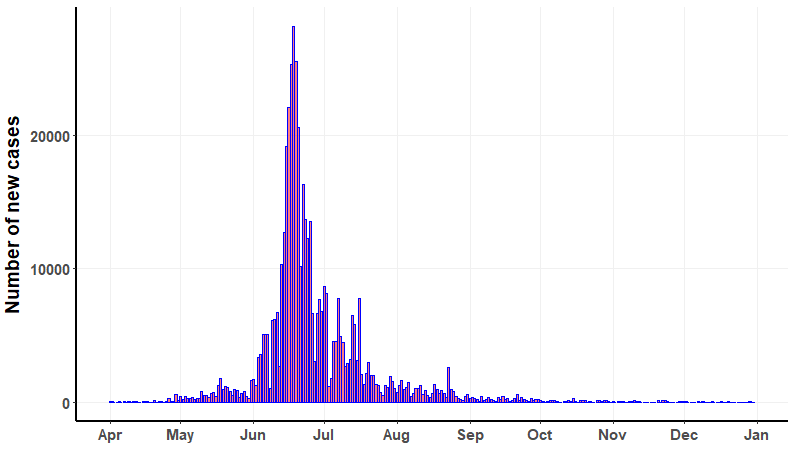


Figure S1. Number of new LSD cases on daily basis in Thailand from April to December, 2021.
